# The clinical characteristics and risk factors for severe COVID-19 in patients with COVID-19 and tuberculosis coinfection

**DOI:** 10.3389/fmicb.2022.1061879

**Published:** 2022-12-22

**Authors:** Yang Wang, Yanping Chen, Lina Gu, Lixin Lou, Jian Zhang, Kaiyu Zhang

**Affiliations:** ^1^Center of Infectious Disease and Pathogen Biology, Department of Infectious Diseases, The First Hospital of Jilin University, Changchun, China; ^2^Department of Tuberculosis, Changchun Infectious Disease Hospital, Changchun, China; ^3^Intensive Care Unit, The First Hospital of Jilin University, Changchun, China; ^4^Department of Infectious Diseases, Changchun Infectious Disease Hospital, Changchun, China

**Keywords:** COVID-19, tuberculosis, coinfection, epidemiological, clinical characteristics, RNA negative conversion time

## Abstract

**Background:**

Under the wave of the severe acute respiratory syndrome-coronavirus-2 (SARS-CoV-2) variant Omicron epidemic, the number of infectious cases has increased dramatically in Jilin Province, China since March 2022.The clinical features and severity of SARS-CoV-2 Omicron variant infection in tuberculosis (TB) patients are not yet clear.

**Methods:**

Data were obtained from 153 patients with the Omicron variant and TB coinfection and 153 non-TB COVID-19 patients who had been hospitalized at Changchun Infectious Disease Hospital from March to June 2022.

**Results:**

Among these coinfection patients, 17 patients showed COVID-19-related pneumonia on chest imaging and 11 were diagnosed with severe COVID-19. The median duration of SARS-CoV-2 clearance was 13 days. The negative conversion time was associated with age, COVID-19-related pneumonia and antibody IgG. A higher white blood cell count, a lower lymphocyte percentage, a higher CRP level, and a higher D-dimer level were found in the severe group. Age and increased PCT were individual risk factors for the severity of COVID-19. Compared with the non-TB patients, the coinfection patients had higher severity of COVID-19 and the elder coinfection patients had a longer negative conversion time.

**Conclusion:**

This study found an association between age, pneumonia, antibody IgG and RNA negative conversion time in COVID-19 and TB coinfection patients, and age and increased PCT were risk factors for the severity of COVID-19.

## Introduction

Coronavirus disease 2019 (COVID-19), caused by severe acute respiratory syndrome coronavirus 2 (SARS-CoV-2), has quickly spread around the world (Lai et al., 2020).To adapt to human hosts, SARS-CoV-2 has been prone to genetic evolution with the development of mutations over time, resulting in mutant variants that may have different characteristics than its ancestral strains (Aleem et al., 2022). Several variants of SARS-CoV-2 have been described by the World Health Organization (WHO), especially given their impact on global public health. Alpha, Beta, Gamma, Delta, and Omicron have been identified as the five SARS-CoV-2 variants of concern (VOCs) since the beginning of the pandemic (Hirabara et al., 2021). The SARS-CoV-2 Omicron variant was first reported from Botswana on November 11, 2021, and it has now replaced the other variants as the main epidemic strain, and this strain is characterized by multiple mutations, stronger infectivity, immune escape characteristics, and a lower risk of severe disease (Karim and Karim, 2021).Under the wave of the Omicron epidemic, the number of infectious cases has increased dramatically in Jilin Province, China from March 2022 (Fan et al., 2022; Li et al., 2022a,b).

The WHO estimated that 9.9 million people fell ill with tuberculosis (TB) worldwide in 2020, and TB is always one of the most important public health concerns in the world. China has the second-highest number of TB cases (8.5%) in the world and is still one of the countries with the highest TB burden (Long et al., 2021). In the past 2 years, the COVID-19 pandemic has caused enormous health, social and economic impacts, including the provision of and access to essential TB services, the number of people diagnosed with TB and the notifications of TB cases through the national disease surveillance systems and TB disease burden (Umubyeyi Nyaruhirira et al., 2022). While the experience of COVID-19 infection in TB patients remains limited, it is anticipated that people ill with both TB and COVID-19 may have poorer COVID-19 treatment outcomes, and TB should be considered a risk factor for severe COVID-19 (TB/COVID-19 Global Study Group, 2022). In a previous study, there were high cases of COVID-19 infections in countries that had less BCG vaccination as compared to countries that immensely use BCG vaccine. It indicated that those vaccinated against TB have been found to have lower susceptibility to COVID-19 (Mariita and Musila, 2020). To report the available evidence on the interaction between COVID-19 and TB, especially for the Omicron variant coinfection, we retrospectively reviewed the epidemiological and clinical manifestations of 153 hospitalized patients with COVID-19 and TB coinfection at Changchun infectious disease hospital (Changchun, China) during March 2022–June 2022.

## Materials and methods

### Ethics statement

Ethical approval for this study was obtained from the Ethics Committee of Changchun Infectious Disease Hospital and the Ethics Committee of the 1^st^Hospital of Jilin University, China. Before data analysis and reporting, all personal identifiers of the patients were removed. All patients provided written informed consent.

### Study population and data collection

In this study, hospital COVID-19 and TB coinfection admissions at Changchun Infectious Disease Hospital (Changchun, China) from March 2022 to June 2022 during the outbreak were involved. To compare the time of SARS-CoV-2 RNA negative conversion, a group of COVID-19 patients without TB who were hospitalized in the same hospital during the same period was chosen as a control, who were chosen with a 1:1 ratio (*n* = 153) and matched for age and gender. The inclusion criteria for COVID-19 and TB coinfection patients included the following: (1) age ≥ 14 years; (2) diagnosis of COVID-19 by confirmed SARS-CoV-2 PCR results in accordance with Chinese Guidelines for Prevention and Control of Novel Coronavirus (Edition 9; CDC, 2022); (3) TB patients who were diagnosed in accordance with the Chinese national guidelines for the prevention and control of TB. Several tests are used to diagnose TB, including acid-fast bacilli smear, GeneXpert, culture, a tuberculin skin test (TST), interferon-gamma release assays and computer tomography (CT); and (4) patients who complied with the study procedures and agreed to participate in the study. All the patients’ records were reviewed for admission, clinical severity, medical history, vaccination, and prognosis. All patients were included in the sample.

### Statistical analysis

Descriptive statistics are presented as the mean ± standard deviation (SD), median (interquartile range), or number (percentage) for continuous variables and frequencies for categorical variables. Comparisons between the groups were analyzed with a 2-tailed Student’s *t* test for parametric analysis and a Mann–Whitney *U* test for nonparametric data analysis. Categorical variables are presented as counts and percentages, and differences between the groups were analyzed with a Pearson *χ*^2^ test or Fisher’s exact test to assess categorical variables. Univariate logistic regression analysis with a *p* < 0.1. Then, multivariate logistic regression analysis was performed on the indicators with *p* < 0.05 in the univariate logistic regression analysis. Statistical analyses were performed using SPSS ver. 22.0 (SPSS, Inc., Chicago, IL, United States). A *p* < 0.05 (two-sided) was considered statistically significant.

## Results

### Demographic, epidemiological, and clinical characteristics

**Figure 1:**
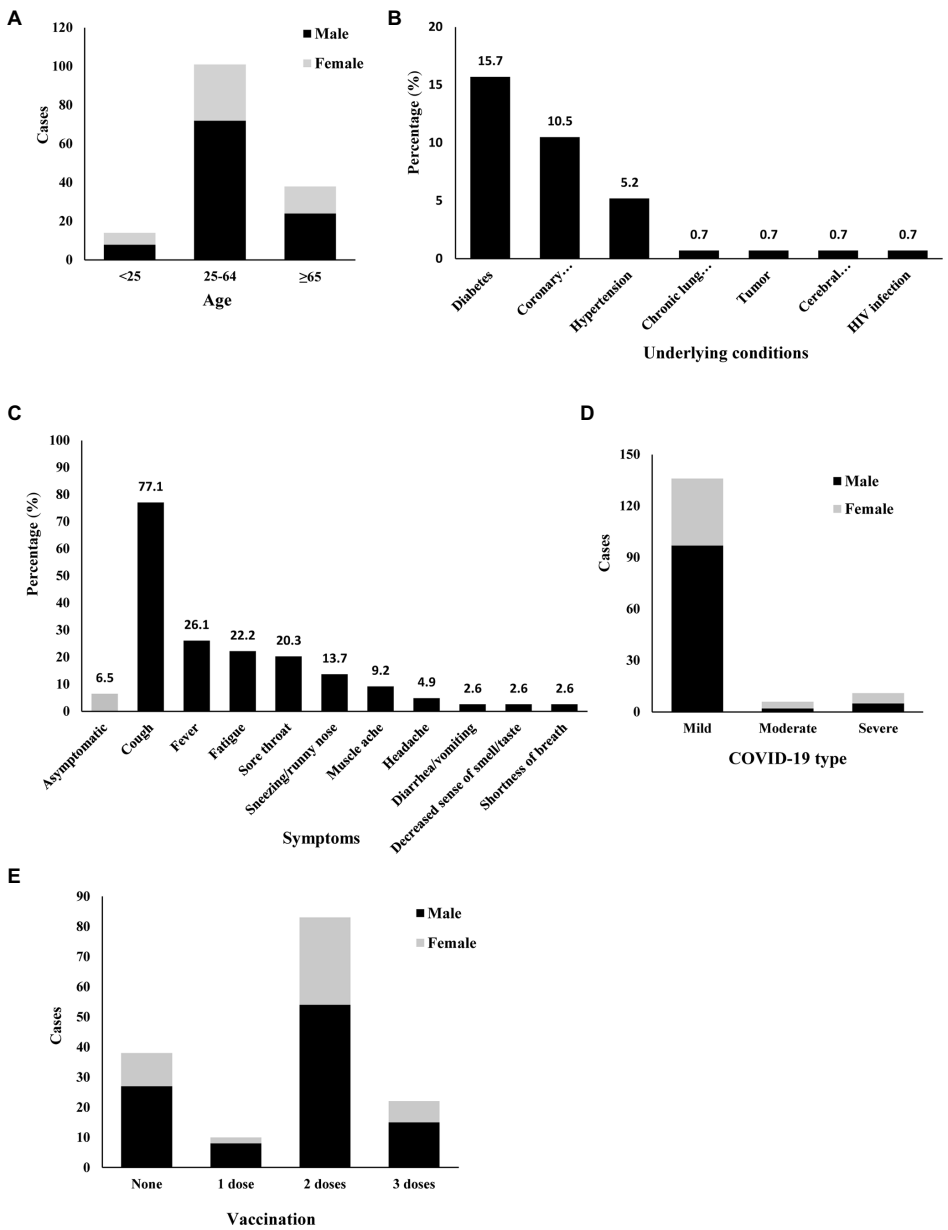
Demographic, epidemiological, and clinical characteristics of 153 COVID-19 and TB coinfection patients. Diabetes, Coronary heart disease, Hypertension, Chronic lung disease, Tumor, Cerebral infarction, HIV infection. **(A)** Age, **(B)** Underlying conditions, **(C)** Symptoms, **(D)** COVID-19 type, **(E)** Vaccination.

A total of 153 patients (104 males and 49 females) were enrolled in this study. The age of these patients (median [interquartile range]) was 53 (41–64). The youngest patient was 15 years old, and the oldest patient was 89 years old. Patients aged 65 and older counted for 24.8% (Figure 1A). Forty-five patients had chronic diseases, including diabetes, coronary heart disease, hypertension, chronic lung disease, tumors, cerebral infarction, and HIV infection. The most common comorbidity was diabetes (*n*, %; 24, 15.7%), followed by coronary heart disease (16, 10.5%), and hypertension (15, 10.5%; Figure 1B).

One hundred and twenty-nine patients had active TB, and 124 were under TB treatment. Five active TB patients were not under TB treatment. Among them, one did not start TB treatment because of his abnormal liver function. The other four refused TB treatment because of the side effects and long course of anti-TB drugs. Among the 124 patients undergoing anti-TB treatment, the diagnosis of COVID-19 was made during months 1–2 for 70 (56.5%) patients, during months 3–6 for eight (6.5%) patients, during months 7–12 for seven (5.6%) patients, and after 12 months for 39 (31.5%) patients. The longest TB history of the active TB patients was 30 years. One hundred and eight patients had pulmonary TB only, and 21 had both pulmonary TB and extrapulmonary TB. Twenty-four patients only had extrapulmonary TB without lung involvement. Among the extrapulmonary TB cases, pleural TB and bone TB were the most common. Among the 129 active TB patients, 12 patients (7.8%) had drug-resistant TB. In China, the BCG vaccine is part of the legal immunization program. China began to enforce BCG vaccination in 1949. After 1978, the planned immunization policy was well implemented. In the past 20 years, the BCG vaccination rate of newborns in Jilin Province was higher than 90%. Through history taking, most patients did not remember whether they had been vaccinated with BCG. So the percentage of BCG immunized history was not involved here.

Only 10 patients had no symptoms related to COVID-19. In these symptomatic coinfection patients, the major symptoms related to COVID-19 included cough, fever, fatigue, sore throat, sneezing/runny nose, muscle ache, headache, diarrhea/vomiting, decreased sense of smell/taste, and shortness of breath. Cough (77.1%) and fever (26.1%) were the most common symptoms (Figure 1C).

Radiological information was available for all patients (100%). Only 17 patients (11.1%) had chest high-resolution computed tomography (CT) findings highly suggestive of COVID-19-related pneumonia (bilateral ground-glass opacities). Among these 17 patients, 11 were diagnosed with severe COVID-19 (oxygen saturation ≤ 93% in the resting state or arterial partial pressure of oxygen/inhaled oxygen concentration ≤ 300 mmHg), while the other 6 patients with typical COVID-19-related changes were diagnosed with moderate COVID-19. All patients without typical COVID-19-related changes (136, 88.9%) were diagnosed with mild COVID-19, and their oxygen saturation was more than 95% (Figure 1D).

Most patients (68.6%) had more than 2 doses of a vaccine (inactivated COVID-19 vaccine), and 22 (14.4%) received three doses. However, 10 (6.5%) received only a single dose of vaccine, and 38 (24.8%) were still unvaccinated (Figure 1E). We compared the symptoms between fully more than fully vaccinated and less than 2-dose vaccinated patients. Full vaccination patients had lower incidence of cough (Supplementary Table S1). Because of the Chinese government’s prevention and control policy for COVID-19, all the patients were hospitalized. Nirmatrelvir plus ritonavir (Paxlovid) is available in China (CDC, 2022). However, Paxlovid was not used in these COVID-19 and TB coinfection patients since it could not be used in combination with rifampin. The patients with severe COVID-19 were given oxygen therapy. Two of them needed ICU admission. No specific anti-COVID-19 treatment was used for any of these patients. Chinese traditional medicine was given to most patients who prefer to be accepted. No patient died by the end of the study.

### Laboratory characteristics

SARS-CoV-2 antibody tests were performed for all patients on admission. The IgM antibody result of seven patients (4.6%) was positive, and the immunoglobulin G (IgG) antibody result of 32 patients (20.9%) was positive. All 153 patients were tested by SARS-CoV-2 PCR every day from admission for confirmation of SARS-CoV-2 infection. The median duration of viral clearance was 13 days (10, 21). The shortest time was 2 days, and the longest time was 61 days. We investigated factors associated with the negative conversion time of viral RNA (Table 1). The negative conversion time in the old age group (≥ 65) was significantly longer than that in the young age and middle age groups [<65; 19.5 (12, 31) vs. 13 (9, 15), *p* = 0.002]. We also compared the data between the patients without COVID-19-related CT changes (the mild type of COVID-19) and the patients with those typical changes (the moderate and severe types of COVID-19). The RNA negative conversion time in the patients with COVID-19-related CT changes was significantly longer than that in those without CT typical changes [25 (12, 36.5) vs. 13 (10, 19), *p* = 0.007]. The patients with positive antibody IgG had an obviously shorter RNA negative conversion time than the patients with negative antibody IgG [10 (5, 15) vs. 14 (11, 21), *p* = 0.046]. Sex, comorbidities, symptoms, COVID-19 vaccination and the site of TB did not appear to affect the time of SARS-CoV-2 RNA negative conversion. However, when the mean duration of viral clearance was counted, the time in the 3-dose vaccination group was shorter than that in the less than 3-dose shot group (13.55 ± 7.63 days vs. 17.48 ± 11.62 days), and the time in the single-dose vaccination and nonvaccination groups was even longer (19.66 ± 14.04 days).

**Table 1:** The duration of SARS-CoV-2 clearance in 153 COVID-19 and TB coinfection patients.

Factors	Count (%)	Negative conversion Time			χ^2^	*p*
		<13 days	13–20 days	≥21 days		
Overall	153	64	48	41		
**Gender**						
Female	49	24	12	13	0.594	0.441
Male	104	40	36	28		
**Age**						
<65	115(75.2%)	54	38	23	9.854	**0.002**
≥65	38(24.8%)	10	10	18		
**Symptom**						
Asymptomatic	10 (6.5%)	5	3	2	0.359	0.549
Symptomatic	143 (93.5%)	59	45	39		
**COVID-19 related CT changes**						
Yes	17(11.1%)	4	3	10	7.251	**0.007**
No	136(88.9%)	60	45	31		
**COVID-19 type**						
Nonsevere	142(88.9%)	61	46	35	3.175	0.075
Severe	11(7.2%)	3	2	6		
**Antibody IgG**						
Positive	32(20.9%)	19	7	6	3.967	**0.046**
Negative	121(79.1%)	45	41	35		
**Comorbidities**						
Yes	45(29.4%)	18	17	10	0.072	0.789
No	108(70.6%)	46	31	31		
**≥2-dose COVID-19 vaccination**						
Yes	105(68.6%)	44	39	22	1.756	0.185
No	48(31.4%)	20	9	19		
**3-dose COVID-19 vaccination**						
Yes	22(14.4%)	10	9	3	1.084	0.298
No	131(85.6%)	54	39	38		
**TB activity**						
Active	129(84.3%)	53	38	38	1.427	0.232
LTBI	24(15.7%)	11	10	3		
**TB Site**						
Pulmonary TB	99(64.7%)	17	63	97	1.956	0.162
Extrapulmonary TB	24(15.7%)	1	16	23		
Pulmonary-extrapulmonary TB	20(13.1%)	2	11	18		

LTBI, Latent tuberculosis infection. Categorical variables are presented as counts and percentages, and differences between groups were analyzed with a Pearson χ^2^ test to assess categorical variables. *p* < 0.05 was considered statistically significant. The bold *p* values were considered statistically significant.

Routine blood tests, liver function tests, and kidney function tests were performed for all patients on admission (Table 2). We compared the results between the nonsevere COVID-19 and severe COVID-19 groups. As demonstrated in Table 3, a higher white blood cell count (*p* = 0.049), a lower lymphocyte percentage (*p* = 0.032), and a higher C reactive protein (CRP; *p* = 0.001) was observed in the severe group than in the nonsevere group. The procalcitonin (PCT) level in the severe group also seemed higher, but the differences were not significant (*p* = 0.104). The D-dimer level in the severe group was significantly higher than that in the nonsevere group (*p* = 0.002).

**Table 2:** The routine laboratory data of 153 coinfection patients [median (interquartile range)].

Variables	Nonsevere group (*n* = 142)	Severe group (*n* = 11)	*Z*	*p*
WBCs (×10^9^/L)	4.85 (3.68, 6.00)	5.40 (5.10, 9.00)	−1.968	**0.049**
Lymphocytes (×10^9^/L)	1.40 (1.00, 1.73)	1.10 (0.90, 1.80)	−0.821	0.412
Lymphocytes (%)	28.44 (22.28, 40.00)	20.75 (10.11, 34.62)	−2.144	**0.032**
HGB (g/L)	137.5 (121.8, 147.3)	132.0 (113.0, 141.0)	−1.201	0.230
PLT (×10^9^/L)	201.5 (149.5, 256.0)	189.0 (125.0, 248.0)	−0.611	0.541
CRP (mg/L)	13.62 (4.56, 32.05)	34.99 (25.56, 148.30)	−3.384	**0.001**
PCT (ng/L)	0.08 (0.05, 0.14)	0.14 (0.10, 0.73)	−2.428	0.104
ALT (U/L)	17.05 (11.98, 30.05)	20.30 (7.90, 58.20)	−0.502	0.616
AST (U/L)	22.00 (17.18, 31.93)	31.10 (18.40, 49.60)	−1.826	0.068
CRE (umol/L)	56.78 (45.41, 65.70)	60.74 (39.80, 80.22)	−0.662	0.522
D-dimer (mg/L)	0.42 (0.24, 0.94)	1.51 (0.93, 2.86)	−3.143	**0.002**
FBG (g/L)	3.88 (3.16, 5.01)	4.38 (3.30, 5.50)	−0.629	0.529
APTT (s)	32.50 (30.45, 34.95)	32.10 (30.90, 35.60)	−0.573	0.567
PT (s)	13.30 (12.60, 14.00)	14.00 (12.80, 14.70)	−1.626	0.104
TT (s)	16.50 (15.25, 17.65)	16.30 (14.50, 16.70)	−1.291	0.197

WBCs, white blood cells; HGB, hemoglobin; PLT, platelet; CRP, C reactive protein; PCT, procalcitonin; ALT, alaninetransaminase; AST, aspartate aminotransferase; CRE, creatinine; FBG, fibrinogen; APTT, active partial thromboplastin time; PT, prothrombin time; TT, thrombin time. All data were nonnormally distributed. Comparisons between groups were analyzed with a Mann–Whitney U test. *p* < 0.05 was considered statistically significant. The bold *p* values were considered statistically significant.

**Table 3:** Univariate analysis of risk factors associated with severe COVID-19.

	Nonsevere group [*n*(%)]	Severe group [*n*(%)]	*χ* ^2^	*p*
**Gender**				
Female	43 (87.8%)	6 (12.2)	2.743	**0.098**
Male	99 (95.2)	5 (4.8)		
**Age**				
<65	112 (97.4)	3 (2.6)	14.466	**<0.001**
≥65	30 (78.9)	8 (21.1)		
**Symptom**				
Asymptomatic	10 (100.0)	0 (0.0)	0.823	0.364
Symptomatic	132 (92.3)	11 (7.7)		
**TB activity**				
Active	119 (92.2)	10 (7.8)	0.387	0.534
LTBI	23 (95.8)	1 (4.2)		
**TB Site**				
Pulmonary TB	101 (93.5)	7 (6.5)	1.995	0.369
Extrapulmonary TB	23 (95.8)	1 (4.2)		
Pulmonary-extrapulmonary TB	18 (85.7)	3 (14.3)		
**Comorbidities**				
Yes	41 (91.1)	4 (8.9)	0.274	0.601
No	101 (93.5)	7 (6.5)		
**AntibodyIgG**				
Positive	31 (96.9)	1 (3.1)	0.995	0.318
Negative	111 (91.7)	10 (8.3)		
**≥2-dose COVID-19 vaccination**				
Yes	99 (94.3)	6 (5.7)	1.084	0.298
No	43 (89.6)	5 (10.4)		
**3-dose COVID-19 vaccination**				
Yes	22 (100.0)	0 (0.0)	1.977	0.160
No	120 (91.6)	11 (8.4)		
**Decreased LY%**				
Yes	25 (82.6)	4 (13.8)	2.338	0.126
No	117 (94.4)	7 (5.6)		
**Increased CRP**				
Yes	83 (88.3)	11 (11.7)	7.390	**0.007**
No	59 (100.0)	0 (0.0)		
**Increased PCT**				
Yes	41 (83.7)	8 (16.3)	8.960	**0.003**
No	101 (97.1)	3 (2.9)		
**Increased D-dimer**				
Yes	57 (86.4)	9 (13.6)	7.230	**0.007**
No	85 (97.7)	2 (2.3)		
**FBG > 5 g/L**				
Yes	36 (92.3)	3 (7.7)	0.020	0.888
No	106 (93.0)	8 (7.0)		
**PT ↑ > 3 s**				
Yes	3 (75.0)	1 (25.0)	1.940	0.164
No	139 (93.3)	10 (6.7)		

LTBI, Latent tuberculosis infection; LY, lymphocytes; CRP, C-reactive protein; PCT, procalcitonin; FBG, fibrinogen; APTT, active partial thromboplastin time; PT, prothrombin time. Categorical variables are presented as counts and percentages, and differences between the groups were analyzed with a Pearson *χ*^2^ test or Fisher’s exact test to assess categorical variables. *p* < 0.1 was considered statistically significant. The bold *p* values were considered statistically significant.

### Univariate and multivariate analysis of risk factors for the severity of COVID-19

The univariate logistic regression analysis showed that older age, female sex, and increased CRP, PCT and D-dimer were associated with the severity of COVID-19 (Table 3). Some previous reports regarded an increased D-dimer level as a risk factor for the severity of COVID-19. However, different cutoff values for D-dimer were used (Simadibrata and Lubis, 2020). The most common cutoff values of D-dimer were 0.5 and 1 μg/ml. We used these two cutoff values in the univariate logistic regression analysis and found that both were associated with the severity of COVID-19. In Table 3, a cutoff value of 0.5 μg/ml was used in the data for D-dimer. All variables with significant differences in the univariate logistic regression analysis were used to construct a multivariable logistic regression model. In multivariable analysis, age ≥ 65 years (OR 9.550; 95% CI 2.283–39.949; *p* = 0.002) and increased PCT (OR 6.253; 95% CI 1.483–26.367; *p* = 0.013) were individual risk factors for the severity of COVID-19.

### Differences between COVID-19 and TB coinfection cases and non-TB COVID-19 cases

We chose a group of COVID-19 patients without TB to investigate the influence of TB infection on RNA negative conversion time and the severity of COVID-19. Control cases were chosen with 1:1 ratio and matched for age and gender. No specific treatment for COVID-19 was used. We compared the negative conversion time and COVID-19 vaccination status between coinfection cases and non-TB cases (Table 4). There’s no significant difference (*p* = 0.392). However, the negative conversion time of elder cases in coinfection group was much longer than that in non-TB group (*p* = 0.040). Among the 153 non-TB cases, 11 with typical COVID-19-related changes were diagnosed with moderate COVID-19, and no case was diagnosed with severe COVID-19. The severity rate of coinfecion group was much higher than that of control group (*p* = 0.001). There was no significant difference in full doses of COVID-19 vaccination between the coinfection group and the control group (*p* = 0.095). But the ratio of more than 2-dose vaccination of the coinfection group was lower than the control group (68.6% vs. 77.1%).

**Table 4:** Differences between COVID-19 and TB coinfection cases and non-TB COVID-19 cases.

	Coinfection cases	Non-TB cases	*Z* or *χ*^2^	*p*
**RNA Negative conversion time (days)**				
Total	13 (10, 21)	14 (10, 18)	−0.855	0.392
<65 [median (IQR)]	13 (9, 16)	14 (10, 18)	−1.524	0.128
≥65 [median (IQR)]	19 (12, 31)	14 (11, 22)	−2.049	**0.040**
**Type**				
Nonsevere[n (%)]	142 (92.8)	153 (100.0)	11.41	**0.001**
Severe[n (%)]	11 (7.2)	0 (0.0)		
**≥2-dose COVID-19 vaccination**				
Yes[n (%)]	105 (68.6)	118 (77.1)	2.794	0.095
No[n (%)]	48 (31.4)	35 (22.9)		

IQR, interquartile range. All data were nonnormally distributed. Comparisons between groups were analyzed with a Mann–Whitney U test. *p* < 0.05 was considered statistically significant. Categorical variables are presented as counts and percentages, and differences between groups were analyzed with a Pearson *χ*^2^ test to assess categorical variables. *p* < 0.05 was considered statistically significant. The bold *p* values were considered statistically significant.

## Discussion

Since being declared a global pandemic by the WHO, SARS-CoV-2, the virus responsible for COVID-19, has spread to 223 countries with more than 513 million cases, and more than 6.2 million deaths have been reported globally. The Omicron variant was first documented in the City of Tshwane, Gauteng Province, South Africa, on 9 November 2021 and led to exponential increases in the number of cases and a sharp rise in hospital admissions. Because the Omicron variant spreads easily and quickly, the number of new cases increased all over the world after December 2021. Omicron has three lineages or subvariants, BA.1 (B.1.1.529.1), BA.2 (B.1.1.529.2), and BA.3 (B.1.1.529.3). Earlier reports showed that the BA.2 Preliminary data from Denmark and the United Kingdom indicate that the BA.2 variant may be more transmissible than the BA.1 variant (Björk et al., 2022). The subvariant of SARS-CoV-2 is identified as Omicron BA.2 in Changchun (Fan et al., 2022; Li et al., 2022a,b). Compared with Omicron BA.1, Omicron BA.2 has a greater superspreading potential (Guo et al., 2022). Infections with the Omicron variant are relatively milder, and the proportion of asymptomatic patients with Omicron variant infection is much higher than with infections with the former variants. It was reported that the proportion of asymptomatic patients was 36.5–90.7% (Houhamdi et al., 2022; Kim et al., 2022; Zhang et al., 2022). In the latest report from another research team of our hospital, 35% patients with an Omicron variant BA.2 infection were asymptomatic (Li et al., 2022b). The most common symptoms were reported as fatigue and sore throat. Fever and cough are also major symptoms, but only 20% of patients will have fever or cough (Meo et al., 2021; Kim et al., 2022). Given the high transmissibility of the Omicron variant and the large TB infection population, the problem and burden of COVID-19 and TB coinfection patients might it’s more prominent (Dheda et al., 2022; Pai et al., 2022). Among TB patients, we found that the proportion of asymptomatic patients (6.5%) was much lower. The most common symptoms observed in our study were cough (77.1%), followed by fever (26.1%), fatigue (22.2%), and sore throat (20.3%). Although infections with the Omicron variant are considered much milder than infections with the former variant, patients who have coinfections with COVID-19 and TB do not have the mild disease that the general population has.

According to WHO website data, the global vaccine doses administered per 100 population are 148.34. While the Chinese vaccine doses that were administered per 100 population are 228.66 and the proportion of persons fully vaccinated with the last dose of the primary series have reached 85%. Several studies have shown that the Omicron variant can dodge some of the immune protection conferred by previous infection and vaccination, especially in patients who had the inactivated COVID-19 vaccine. However, it has been confirmed by several reports that full-scale vaccination (both mRNA COVID-19 vaccine or inactivated COVID-19 vaccine) could decrease the hospitalization rate, severity rate, and fatality rate of COVID-19 patients (Fan et al., 2021). Sinovac COVID-19 Vaccine and Sinopharm BIBI COVID-19 Vaccine were used in mainland of China and both of them are inactivated COVID-19 vaccine and shared similar technology. Most patients were randomized to inject any of them. Some patients got 2 or 3 doses of the vaccine, but the vaccine was not from the same brand. In our study, 68.6% of COVID-19 and TB coinfection patients received more than two doses of inactivated COVID-19 vaccine. In addition, only 14.4% of the patients received 3 doses of vaccination. More patients (77.1%) received more than two doses of inactivated COVID-19 vaccine in the non-TB COVID-19 group. The vaccination rate in TB patients was obviously lower than that in the general population.

The WHO’s current estimate of the global case fatality rate for the Omicron variant is 0.02–0.31% (Duong et al., 2022). In our study, both the ICU admission rate (1.3%) and fatality rate (0%) of COVID-19 and TB coinfection patients were lower than those reported in Omicron variant-infected patients (Abdullah et al., 2022; Modes et al., 2022). But the severity of COVID-19 in the coinfection group was obviously higher than the non-TB group. The severity and case fatality rate of COVID-19 are affected by many factors. Age, D-dimer, C-reactive protein, sequential organ failure assessment score and body temperature, decreasing albumin, and a history of diabetes were regarded as the major risk factors for COVID-19 severity (Rod et al., 2020). Although the Omicron variant BA.2 has evolvedtoward being less virulent, a higher rate of severe outcomes and mortality have been observed in unvaccinated people, especially older adults (Cheung et al., 2022). Comorbidities are still the major risk factor for hospitalization and death (Zhang et al., 2022). However, in our study, in the patients with COVID-19 and TB coinfection, the patients with underlying medical did not show an increased risk of developing severe COVID-19 infection. The sample size was limited in our study, and more studies with larger samples might show clearer and more definitive conclusions. In our study, older age and increased PCT were individual risk factors associated with the severity of COVID-19.In the elder group, 19 patients (50%) received more than 2 doses of vaccination, and only 3 (7.9%) had 3 shots of the vaccination. Sixteen patients (42.1%) did not receive even a single dose of the vaccination in this group. We also note that among these coinfected patients, all 3-dose vaccinated patients belonged to the nonsevere group, although COVID-19 vaccination did not appear to affect the severity of COVID-19 statistically. Three doses of inactivated COVID-19 vaccine seemed to effectively reduce the severity of COVID-19 in TB patients. In mainland China, the mRNA COVID-19 vaccine is not yet available; therefore, 3-dose inactivated COVID-19 vaccination is recommended in TB patients according to the findings of this study.

Because of the Chinese government’s prevention and control policy for COVID-19, patients could not be discharged from the hospital until the tests for SARS-CoV-2 RNA turned negative. The SARS-CoV-2 PCR test was performed for all patients every day. Therefore, we have a more in-depth understanding of the duration of viral clearance in these patients. In our study, the median duration of viral clearance was 13 days. The shortest time of RNA negative conversion was 2 days, while the longest time was 61 days. Age, COVID-19-related CT changes, and IgG antibodies against SARS-CoV-2 are associated with the duration of viral RNA clearance in TB patients. And the negative conversion time of elder cases in the coinfection group was much longer than that in the non-TB group.

Paxlovid is an antiviral therapeutic being developed by Pfizer for the treatment and postexposure prophylaxis of COVID-19. It has been reported to efficiently reduce the viral load, hospitalization and fatality rate (Lamb, 2022). Given the reported efficacy of Paxlovid against COVID-19, it is recommended to mild or moderate COVID-19 patients within 5 days onset with risk factors of disease progression. Paxlovid is a strong cytochrome P450 (CYP) 3A4 inhibitor and is contraindicated in patients receiving rifampicin, which is a strong CYP3A4 inducer. Because rifampin is one of the first-line agents for treating drug-sensitive TB patients, Paxlovid was not considered for treating TB and COVID-19 coinfection patients. In fact, among the 6 moderate and 11 severe COVID-19 patients, 3 were latent tuberculosis infection patients, and another had rifampin-resistant TB. These four patients did not take rifampin on admission. Among the mild COVID-19 patients, 21 patients were inactive TB patients, 11 patients were rifampin-resistant and 5 active TB patients were not under TB treatment. The application of Paxlovid in tuberculosis patients still needs further individualized evaluation, especially for those patients with risk factors.

Finally, we did not find a higher severity rate or fatality rate in the patients who were infected with both the Omicron variant of COVID-19 and TB in this wave. The variant is milder, and wide vaccination in the population might also play important roles. It is an important task to promote COVID-19 vaccination in TB patients, especially among elderly patients.

## Data availability statement

The raw data supporting the conclusions of this article will be made available by the authors, without undue reservation.

## Ethics statement

Ethical approval for this study was obtained from the Ethics Committee of Changchun Infectious Disease Hospital and the Ethics Committee of the 1stHospital of Jilin University, China. Written informed consent to participate in this study was provided by the participants’ legal guardian/next of kin.

## Author contributions

JZ and KZ: conceptualization and project administration. YW and YC: validation and writing—original draft preparation. YW, LL, and KZ: writing—review and editing. LG: visualization. KZ: supervision. All authors agree to be accountable for the content of the work.

## Funding

This work was supported by the National Natural Science Foundation of China (No. 81801972) to YW, Science and Technology Research Project of Jilin Provincial Department of Education (JKH20211179KJ) to YW, the Jilin Provincial Nature Science Foundation of Jilin Provincial Department of Science and Technology (20210101341JC and 20200201616JC) to YW and KZ, and Jilin Province Health Science and Technology Capacity Improvement Project (2021JC001) to KZ.

## Conflict of interest

The authors declare that the research was conducted in the absence of any commercial or financial relationships that could be construed as a potential conflict of interest.

## Publisher’s note

All claims expressed in this article are solely those of the authors and do not necessarily represent those of their affiliated organizations, or those of the publisher, the editors and the reviewers. Any product that may be evaluated in this article, or claim that may be made by its manufacturer, is not guaranteed or endorsed by the publisher.
