# Supplementary material for: The clinical characteristics and risk factors for severe COVID-19 in patients with COVID-19 and tuberculosis coinfection
Source: Front Microbiol. 2022 Dec 22;13:1061879. doi: 10.3389/fmicb.2022.1061879 (PMC9817148; doi:10.3389/fmicb.2022.1061879)
Supplement: Supplementary file 1 [file Table_1.DOCX]

**STable 1. Comparison of symptoms of COVID-19 between vaccinated and non-vaccinated coinfection patients**

|  | **<2 dose**  **[n(%)]** | | **≥2 dose**  **[n(%)]** | **χ^2^** | ***P*** |
| --- | --- | --- | --- | --- | --- |
| **Symptomatic** |  | |  |  |  |
| Yes | 43 （30.1) | | 100 （69.9) | 0.23 | 0.337 |
| No | 5 （50.0) | | 5 （50.0) |  |  |
| **Cough** |  | |  |  |  |
| Yes | 36 (63.2) | | 21 (36.8) | **40.308** | **0** |
| No | 12 (12.5) | | 84 (87.5) |  |  |
| **Fever** |  | |  |  |  |
| Yes | 12 (30) | | 28 (70) | 0.000 | 0.984 |
| No | 36 (31.9) | | 77 (68.1) |  |  |
| **Fatigue** |  | |  |  |  |
| Yes | 11 (32.4) | | 23 (67.6) | 0.000 | 1 |
| No | 37 (31.1) | | 82 (68.9) |  |  |
| **Sore throat** |  | |  |  |  |
| Yes | 10 (32.3) | | 21 (67.7) | 0.000 | 1 |
| No | 38 (31.1) | | 84 (68.9) |  |  |
| **Sneezing/runny nose** |  | |  |  |  |
| Yes | 9 (42.9) | | 12 (57.1) | 1 | 0.222 |
| No | 39 (29.5) | | 93 (70.5) |  |  |
| **Muscle ache** |  | |  |  |  |
| Positive | 3 (21.4) | | 11 (78.6) | 0.708 | 0.400 |
| Negative | 45 (32.4) | | 94 (67.6) |  |  |
| **Headache** | | |  |  |  |
| Yes | 2 (28.6) | | 5 (71.4) | 0.027 | 0.870 |
| No | 46 (31.5) | | 100 (68.5) |  |  |
| **Diarrhea/vomiting** | | |  |  |  |
| Yes | 1 (25.0) | | 3 (75.0) | 0.077 | 0.781 |
| No | 47 (31.5) | | 102 (68.5) |  |  |
| **Decreased sense of smell/taste** | | |  |  |  |
| Yes | | 1 (25.0) | 3 (75.0) | 0.077 | 0.781 |
| No | | 47 (31.5) | 102 (68.5) |  |  |
| **Shortness of breath** | |  |  |  |  |
| Yes | | 1 (25.0) | 3 (75.0) | 0.077 | 0.781 |
| No | | 47 (31.5) | 102 (68.5) |  |  |

Categorical variables are presented as counts and percentages, and differences between the groups were analyzed with a Fisher's exact test to assess categorical variables. *P*< 0.1 was considered statistically significant.
